# Genetic Parameters for Growth, Ultrasound and Carcass Traits in New Zealand Beef Cattle and Their Correlations with Maternal Performance

**DOI:** 10.3390/ani12010025

**Published:** 2021-12-23

**Authors:** Franziska Weik, Rebecca E. Hickson, Stephen T. Morris, Dorian J. Garrick, Jason A. Archer

**Affiliations:** 1 School of Agriculture and Environment, Massey University, Private Bag 11222, Palmerston North 4442, New Zealand; R.Hickson@massey.ac.nz (R.E.H.); S.T.Morris@massey.ac.nz (S.T.M.); D.Garrick@massey.ac.nz (D.J.G.); 2 AbacusBio Ltd., P.O. Box 5585, Dunedin 9058, New Zealand; 3 Beef + Lamb New Zealand Genetics, P.O. Box 5501, Dunedin 9054, New Zealand; Jason.Archer@blnzgenetics.com

**Keywords:** beef cattle, heritability, genetic correlations, maternal performance, growth, ultrasound, carcass, finishing performance

## Abstract

**Simple Summary:**

Profitability of the beef industry depends on the performance of both finishing cattle and the breeding herd. Breeding programs need to be designed in a way that balances requirements for both systems, requiring knowledge of relationships between trait groups. This study examined the inheritance of growth, ultrasound and carcass traits and their relationships with maternal performance in New Zealand beef herds. Results have shown that genetic variation exists for traits measured in finishing animals such that direct selection for enhanced growth, ultrasound and carcass traits is feasible. Genetic correlations indicate that decreasing fat content in live animals at ultrasound scanning or at slaughter can lead to detrimental changes in the genetic merit of maternal performance. Thus, a reduction in reproductive performance in 2-year-old cows may occur while mature size genotypes are likely to increase, and this can impact on cow maintenance requirements. However, only low genetic correlations exist with body condition score in mature cows such that there is room to alter the fat distribution in finishing animals without impacting on body energy reserves in mature cows. Results indicate that identifying animals with favourable genotypes for both maternal and finishing performance is feasible when measuring animals for both trait groups.

**Abstract:**

Research has shown that enhancing finishing performance in beef cows is feasible; however, any adverse impact of selection strategies for finishing performance on the performance of the maternal herd should be taken into account. The aim of this research was to examine the inheritance of growth, ultrasound and carcass traits in finishing beef cattle and to evaluate their correlations with maternal performance traits. Data were collected from a nationwide progeny test on commercial New Zealand hill country farms comprising a total of 4473 beef cows and their progeny. Most finishing traits were moderately to highly heritable (0.28–0.58) with the exception of meat or fat colour and ossification (0.00–0.12). Ultrasound scan traits had high genetic correlations with corresponding traits measured at slaughter (r_g_ = 0.53–0.95) and may be used as a selection tool for improved genetic merit of the beef carcass. Fat content determined via ultrasound scanning in the live animal or at slaughter in finishing cattle is positively genetically correlated with rebreeding performance (r_g_ = 0.22–0.39) in female herd replacements and negatively correlated with mature cow live weight (r_g_ = −0.40 to −0.19). Low-magnitude associations were observed between the genetic merit for carcass fat traits with body condition in mature cows.

## 1. Introduction

The key driver for performance and profit in a beef cow herd is calf output relative to cow feed requirement [1,2]. The key drivers in a beef finishing system are growth, carcass traits and feed costs [3,4,5]. To improve the performance of beef cattle on pasture, it is relevant to balance the requirements of the entire operation [6]. Breeding programs can incorporate both maternal and finishing traits as selection criteria with different emphasis dependent on the breeding objective [7]. Knowledge of the genetic components that influence maternal and finishing performance and of potential antagonisms between both systems is necessary to enhance breeding strategies, optimize selection decisions [8,9] and ultimately improve the efficiency of the herd [1,10,11].

Many researchers have published genetic and phenotypic parameters for either maternal or finishing traits [9,12] but literature describing antagonisms among those trait groups is sparse. New Zealand’s farming systems rely on extensive grass-fed beef production [13]. Beef cattle are primarily farmed alongside sheep on steeper, less cultivable hill country farms. It is usually impractical to deliver supplementary feed to cows grazed in these systems, and stocking rates are low and determined by the available pasture supply as a result [13]. Different genotypes may be required compared to other systems internationally where cattle are finished on grain [14,15] and/or cows are fed on crop residues [16,17]. Thus, to assess those differences, an understanding of antagonisms among maternal performance, growth, ultrasound and carcass traits based on data from commercial New Zealand farming systems is needed to further investigate relevant traits to be considered for genetic evaluation and breeding program design.

Weik et al. [18] reported genetic parameters of maternal performance including reproduction, live weight, hip height, body condition score and maternal weaning weight traits for the maternal cohort of animals as part of a large-scale nationwide progeny test. The current study builds on that research, further exploring a subset of the maternal traits for the purpose of correlation analysis. The aim of this study was to examine the inheritance of growth, ultrasound and carcass traits measured in finishing beef cattle on commercial New Zealand hill country farms and to evaluate their correlations with key maternal performance traits.

## 2. Materials and Methods

### 2.1. Animal Management

Animal ethics approval was obtained for all related procedures and measures involved in this study (AgResearch Invermay Animal Ethics Committee approval numbers: 13358, 13373, 13394, 13693, 14031, 14311, 14588, 14851, and 15153).

The current study used data collected as part of an ongoing nationwide beef progeny test (BPT) on five large-scale commercial hill country farms in New Zealand. The BPT was established by Beef + Lamb NZ Genetics in 2014 to compare sire performance across a range of commercial environments over multiple years. An in-depth description of the animal management procedures and data collection is provided by Weik et al. [19] and Weik et al. [20]. In summary, the BPT comprises Angus or Hereford breeding cows where some female calves (sired by Angus, Hereford or Stabilizer bulls) were retained as herd replacements and the remaining female and male calves were raised for slaughter. All cattle grazed exclusively on pasture (including some forage crops) and finishing cattle were processed at 18–34 months of age. Heifers within the breeding herd were naturally mated for the first two breeding seasons then cows received a one-off artificial insemination to synchronised oestrus followed by natural mating from their third mating season onwards. Insemination was conducted using semen from a range of New Zealand and international bulls including Angus, Hereford, Charolais, Simmental and the Stabilizer composites over the foundation Angus or Hereford cows, with most bulls in common across herds. Pregnancy diagnosis was conducted approximately 90 days after the start of the mating season and fetal age determined. Birthdate of calves was assigned based on fetal age records, assuming a 282 day gestation [21]. Calving occurred in spring between September and November aligning with the seasonal production cycle typical of extensive beef farming systems in New Zealand.

### 2.2. Measurements and Trait Definitions

Performance records were obtained for a range of traits for females within the breeding herd and for heifers and steers as part of the finishing operation. Female herd replacements were assessed for their maternal performance in terms of reproduction, weight, size and body condition, whereas animals destined for slaughter were measured for growth, ultrasound and carcass traits to examine finishing performance.

Where appropriate, phenotypic records were pre-adjusted to either a constant age, weight, body condition score or hip height at the time of measurement, where pre-adjustments were based on continuous variables using the 4-step procedure of Reverter et al. [22]. Adjustments were made using linear models with contemporary group (CG) as a fixed effect and definitions for CG are presented in the data editing section. Age, weight, body condition score or hip height were fitted as linear or quadratic covariates and pre-adjustments are detailed in the trait descriptions. Quadratic effects of covariates were tested using an F-statistic on type-III sums of squares (analysis of variance) and discarded from the final model via backward elimination if not significant. Adjustments were made on an individual animal basis as opposed to a CG mean to account for within-CG variation.

#### 2.2.1. Maternal Traits

Maternal traits were a subset of those previously described by Weik et al. [18], chosen based on their relevance for the New Zealand beef sector. Rebreeding performance (RB) was defined as pregnancy outcome for 2-year-old cows present at pregnancy diagnosis and was coded as a binary trait (0 = not pregnant, 1 = pregnant). Cows that were not present at their second pregnancy diagnosis were coded as missing. Days to conception (DtC2) describes the time in days from start of the mating season to conception day for 2-year-old cows in their second mating period. Cows that failed to conceive were included in the analysis and assigned a penalty of 21 days after the last conception date within their CG [10,23].

Mature cow live weight (MWT), mature cow body condition score (BCS) and mature cow hip height (MHH) were recorded on cows that were aged 3 years or older. Animals were measured for MWT and BCS at three timepoints throughout the year (mating, weaning, calving) and only one annual measure of MHH was made prior to calving. All three mature cow traits were pre-adjusted to 5 years of age. Age adjustments were made using a fixed-effects model with age (in years) with cows older than 12 years of age grouped in a single group and CG as factors in the model. Scoring BCS was through visual assessment of body energy reserves with 1 being emaciated and 10 being obese [24]. The trait MWT was also pre-adjusted to either a constant BCS of 6 (MWT_BCS_) or a constant hip height of 130 cm (MWT_HH_) to evaluate live weight of cows irrespective of stored energy reserves or size. Adjustments were made using either BCS or hip height as a linear and quadratic covariate, for MWT_BCS_ and MWT_HH_, respectively.

Weaning weight of calves (WWT) was pre-adjusted to a constant 200 days of age using linear models with age (in days) as a covariate.

#### 2.2.2. Growth Traits

Live weights for growing animals were recorded unfasted for WWT between 108 and 228 days of age, yearling weight (YWT) between 272 and 388 days of age and 18 month weight (W18) between 475 and 616 days of age. Weight traits were chosen based on their relevance for the New Zealand beef sector and are commonly recorded traits for growing animals within genetic evaluation programs worldwide [25]. Live weights were pre-adjusted to 200 days for WWT, and to the mean age in the dataset of 344 and 560 days for YWT and W18, respectively. Pre-adjustment models included the covariate of age (in days), and the quadratic effect of age was retained in the model for W18 only. Age-adjusted live weight was used to compute post-weaning gain (PWG) as the difference between YWT and WWT and post-yearling gain (PYG) as the difference between W18 and YWT. Yearling hip height (YHH) was recorded from 2016 onwards and measured at the same time as YWT. A linear model was used to pre-adjust YHH to the mean age at data recording of 351 days, with the linear and quadratic effect of age (in days) as a covariate.

#### 2.2.3. Ultrasound Scan Traits

Ultrasound scanning was conducted on live animals for traits commonly recorded in pedigree-recorded cattle [25], namely eye-muscle area of the longissimus dorsi muscle measured between the 12 and 13th rib (EMA) and the three fat-related traits: intramuscular fat percent (IMF) at the 12/13th rib site, fat depth at the P8 rump site (FD_P8_), and fat depth at the 12/13th rib site (FD_RIB_). Scans were conducted by Breedplan-accredited technicians on replacement heifers and on steers and heifers destined for processing, resulting in records for 1904 steers and 2049 heifers. Recording coincided with W18, between 475 and 616 days of age. All ultrasound traits were pre-adjusted to a constant live weight of 432 kg, the mean live weight at 18 months of age, using linear and quadratic live weight as covariates. This adjustment was used to compare muscularity and fatness rather than absolute size.

#### 2.2.4. Carcass Traits

Following ultrasound scanning, animals were assigned to slaughter management groups based on their live weight. Within these groups, all animals were slaughtered on the same day without further selection prior to slaughter. Measurements on the carcass included carcass weight (CWT), carcass eye-muscle area (CEMA), marbling score (MB), fat colour (FC), meat colour (MC), ossification score (OSS) and rib fat depth (RF) for a total of 1636 steers and 354 heifers and were recorded between 541 and 1029 days of age. The trait CWT was recorded at the time of slaughter, whereas grading measures were assessed by trained graders using the Meat Standards Australia chiller assessment system [26] on the day following slaughter. Measures were taken at the 12/13th rib site for CEMA, MB, FC, MC and RF, whereas the OSS of the carcass was determined on the dorsal spinous processes. A visual score for MB was assigned by the grader. Pre-adjustments were applied for CWT to the average age at slaughter within the dataset of 811 days, whereas all other carcass traits were pre-adjusted to the average CWT in the dataset of 310 kg using age (in days) or weight as linear and quadratic covariates.

### 2.3. Data Editing

#### 2.3.1. Contemporary Group Definitions

Definitions of CG for maternal, growth, ultrasound and carcass traits are outlined in Table 1. Only animals for which all information to form CG was available were considered for analyses, allowing animals that had been treated alike up until the time of measurement to be grouped together. Animals were removed from the dataset when less than one individual was recorded for a CG or when all animals within the same CG had identical values. Following pre-adjustments of the phenotypes, possible outliers were removed from the dataset by deleting any observation further than three standard deviations from the CG mean [27].

#### 2.3.2. Heterogeneous Variances

Traits were tested for departures from homogeneity, and this was evaluated by examining the relationship between the CG mean and CG standard deviation (SD) [28,29]. A linear model was fitted following pre-adjustments by regressing the SD on the mean of the CG. A significant regression coefficient (*p* < 0.05) was considered evidence for the presence of heterogeneous variances, and this was the case for MWT, MWT_BCS_, MWT_HH_, FD_P8_, FD_RIB_, MC and OSS. Consequently, these traits were scaled to homogenize the variances [27] and this was achieved by multiplying each observation by the population mean divided by the mean of the CG of that observation [30].

Each trait included in this study with number of records, range of data measure, mean and SD is presented in Table 2.

### 2.4. Statistical Analysis

Data cleaning and formatting were conducted using R version 3.6.1 [31]. Restricted maximum likelihood was used to estimate (co)variance components fitting various animal models in the ASREML 4.1 software package [32].

Variance components were obtained for all growth and ultrasound traits using univariate animal models. For carcass traits, variance components were estimated using bivariate models including W18 to account for any selection that occurred prior to data recording due to drafting of animals in lighter and heavier slaughter mobs. Genetic and phenotypic correlations were obtained among all maternal and finishing traits for which heritability was greater than 0.05 by estimating (co)variance components from bivariate animal models, or trivariate models (when a carcass trait and W18 were included).

Models included various fixed effects which were not accounted for through pre-adjustments of the phenotypes. Those along with random effects fitted for each trait are shown in Table 3. The random effects included in each model were based primarily on relevance for each trait and data availability. Those traits measured in mature cows had repeated records over time (within and across years) such that a permanent environmental effect was included in the analysis to account for sustained data recording. Maternal grandsire and granddam information was available for progeny from naturally-mated heifers that were recorded up until weaning, allowing a maternal genetic effect to be included in the analysis for WWT. Additionally, a permanent environmental effect of the dam was included for WWT to account for dams with multiple calves across years.

The general models used can be described as follows:

(1) $$y_{\mathrm{WWT}}=Xb+Z_{1}{a+Z}_{2}{m+Z}_{3}\mathrm{me}+\varepsilon$$

Or

(2) $$y_{\mathrm{repeat}}=Xb+Z_{1}{a+Z}_{2}\mathrm{pe}+\varepsilon$$

Or

(3) $$y_{\mathrm{single}}=Xb+Z_{1}a+\varepsilon ,$$

where $y_{\mathrm{WWT}}$ was the vector of pre-adjusted phenotypic records for maternally-influenced WWT, $y_{\mathrm{repeat}}$ was the vector of pre-adjusted phenotypic records for traits with repeated measures such as MWT and included a term for the permanent environmental effect (pe) of the animal to account for correlated residuals, $y_{\mathrm{single}}$ was the vector of pre-adjusted phenotypic records for traits that were not maternally-influenced and comprised only a single phenotypic observation such as PWG, the terms X and Z were incidence matrices relating vectors of fixed effects (b) and random direct additive-genetic (a), maternal genetic (m), maternal permanent environmental (me) and permanent environmental (pe) effects to each observation in y and ε was the vector of residual effects.

The expected values of y and (co)variance structures for the random effects among traits were assumed as follows:

(4) $$E\left[\begin{array}{c} y\\ a\\ \begin{array}{c} m\\ \mathrm{me}\\ \varepsilon \end{array} \end{array}\right]=\left[\begin{array}{c} \mathrm{Xb}\\ 0\\ 0\\ 0\\ 0 \end{array}\right];\;\mathrm{var}\left[\begin{array}{c} a\\ \begin{array}{c} m\\ \mathrm{me}\\ \varepsilon \end{array} \end{array}\right]=\left[\begin{array}{cccc} A\;⊗\;G & A\;⊗\;d & 0 & 0\\ A\;⊗\;d & {A\sigma }_{m}^{2} & 0 & 0\\ 0 & 0 & {I\sigma }_{\mathrm{me}}^{2} & 0\\ 0 & 0 & 0 & I\;⊗\;E \end{array}\right]$$

for bivariate or trivariate models with WWT (model Equation (1)) and one or two single observation traits (model Equation (3));

(5) $$E\left[\begin{array}{c} y\\ a\\ \begin{array}{c} \mathrm{pe}\\ \varepsilon \end{array} \end{array}\right]=\left[\begin{array}{c} \mathrm{Xb}\\ 0\\ 0\\ 0 \end{array}\right];\;\mathrm{var}\left[\begin{array}{c} a\\ \begin{array}{c} \mathrm{pe}\\ \varepsilon \end{array} \end{array}\right]=\left[\begin{array}{ccc} A\;⊗\;G & 0 & 0\\ 0 & {I\sigma }_{\mathrm{pe}}^{2} & 0\\ 0 & 0 & I\;⊗\;E \end{array}\right]$$

for bivariate or trivariate models with one trait containing a permanent environmental effect of the animal (model Equation (2)) and one or two single observation traits (model Equation (3)); and

(6) $$E\left[\begin{array}{c} y\\ a\\ \begin{array}{c} \varepsilon \end{array} \end{array}\right]=\left[\begin{array}{c} \mathrm{Xb}\\ 0\\ 0 \end{array}\right];\;\mathrm{var}\left[\begin{array}{c} a\\ \begin{array}{c} \varepsilon \end{array} \end{array}\right]=\left[\begin{array}{cc} A\;⊗\;G & 0\\ 0 & I\;⊗\;E \end{array}\right]$$

for bivariate or trivariate models with single observation traits (model Equation (3)). In these model descriptions, A  was the numerator relationship matrix, G was the genetic (co)variance matrix (2 × 2 or 3 × 3), d was a vector of covariances between maternal effects for WWT and additive-genetic effects for other traits (2 × 1 or 3 × 1), $\sigma_{m}^{2}$ was the variance due to maternal genetic effects for WWT, I  was an identity matrix of the order equal to the number of animals with observations, $\sigma_{\mathrm{me}}^{2}$ was the variance due to maternal permanent environmental effects for WWT, $\sigma_{\mathrm{pe}}^{2}$ was the variance due to permanent environmental effects of the animal, E was a residual (co)variance matrix (2 × 2 or 3 × 3; for traits not expressed within the same animal (maternal and carcass traits) no residual covariances were identifiable and residual covariances were constrained to be zero in the ASREML model), and ⊗ denotes the Kronecker product operator.

Convergence was presumed according to the default value in ASREML when the log likelihood changed less than 0.002-fold the number of iterations and the change of individual variance parameter estimates was below 1%. The pedigree was traced back for up to two generations so that the numerator relationship matrix A included a total of 14,240 individual animals including 424 sires and 4473 dams. Ancestral pedigree was not available for founder cows or sires and the base population was assumed unrelated.

## 3. Results

### 3.1. Variance Components and Heritabilities

Results from univariate animal models for the estimation of variance components and heritabilities for growth and ultrasound traits as well as from bivariate analysis for carcass traits are presented in Table 4.

Estimates for heritabilities were moderate for the growth traits PWG and PYG and ranged from 0.33 to 0.35. Heritabilities were higher for both weight-related traits YWT and W18 with 0.53 and 0.50, respectively. Variance components increased for both additive-genetic and residual variances when estimated for the same trait in older compared to younger animals. The trait YHH was the most heritable growth trait at 0.55.

Weight-constant ultrasound traits were moderately to highly heritable, and the lowest heritability estimate was for EMA (0.36). Estimates of heritability were generally greater among those traits related to fat deposition, namely IMF, FD_P8_ and FD_RIB_ (0.52–0.58), but variances were lower than for EMA.

Heritability estimates varied considerably across traits measured on the carcass at slaughter and were generally low to moderate. The most heritable carcass trait was CWT with 0.47 estimated at a constant age. Moderate heritabilities were also observed for the weight-constant traits CEMA (0.43) and MB (0.39). All other carcass traits were only lowly heritable and ranged from 0.07 to 0.28 for FC, OSS and RF. The trait MC was not heritable and variance components were low for both traits related to colour of the carcass (MC and FC).

### 3.2. Correlations among Finishing Traits

Genetic and phenotypic correlations among traits measured in finishing cattle are shown in Table 5.

Weight traits of growing animals generally had high genetic correlations (0.70–0.88) among themselves, but correlations with the growth-related traits PWG (0.08–0.18) and PYG (0.10–0.68) were overall lower. Those traits related to fat deposition measured at ultrasound scanning were highly correlated and ranged from 0.68 to 0.85 among IMF, FD_P8_ and FD_RIB_. Both, FD_P8_ and FD_RIB_ were not correlated with EMA (0.06), but a moderate genetic correlation has been observed between EMA and IMF (0.36). Genetic correlations among carcass traits ranged from a highly negative correlation between CWT and FC (−0.63) to a high positive correlation between CEMA and FC (0.73).

The trait CWT was only lowly genetically correlated with weight-constant ultrasound traits and ranged from −0.06 to 0.14. High genetic correlations were obtained among corresponding traits measured at either ultrasound scanning or at slaughter for EMA and CEMA (0.53), IMF and MB (0.70) and FD_RIB_ and RF (0.95). Overall, traits related to the fat content of live animals at ultrasound scanning (IMF, FD_P8_ and FD_RIB_) compared to carcass traits (MB and RF) were moderately to highly genetically correlated and ranged from 0.38 to 0.95. Low genetic correlations were generally obtained for OSS or FC with all traits measured in live animals at ultrasound scanning (−0.19–0.23), but a moderate genetic correlation was observed between FC and EMA (−0.34).

Generally, phenotypic correlations were lower compared to genetic correlations among all traits measured in finishing animals. The highest phenotypic correlations were estimated among weight traits such as YWT, W18 and CWT and ranged from 0.56 to 0.77. Low to high phenotypic correlations were observed among traits related to fat deposition in either live animals at ultrasound scanning or at slaughter and ranged from 0.22 to 0.72.

### 3.3. Genetic Correlations among Maternal and Finishing Traits

Genetic correlations among maternal performance and finishing traits are presented in Table 6.

Genetic correlations of the reproductive traits RB and DtC2 with finishing traits were similar but with opposing signs except for PYG which was positively correlated with both reproduction traits. Traits related to growth in yearling animals (PWG, YWT and YHH) were positively genetically correlated with RB in the current study but correlations were low (0.11–0.18). Estimates were slightly greater for growth traits measured in older animals (PYG and W18) and ranged from 0.22 to 0.32. Moderate genetic correlations were estimated among reproductive traits and ultrasound scan traits related to fat deposition such as IMF, FD_P8_ and FD_RIB_ and those correlations were positive for RB (0.31–0.39) and negative for DtC2 (−0.42 to −0.35). Correlations among fat-related traits measured on the carcass at slaughter (MB, RF) were slightly lower for RB (0.22–0.36) but in a similar range for DtC2 (−0.59 to −0.29). The trait RB was lowly positively (0.11) correlated with EMA at ultrasound scanning after the effect of weight has been accounted for but was moderately negatively correlated with the corresponding trait measured at slaughter (−0.46).

Generally, MWT was highly genetically correlated with most growth traits, ranging from 0.62 to 0.92, but only a moderate correlation was observed with PWG (0.45). Similarly, the genetic correlation with CWT was high (0.82). Genetic correlations of MWT with all ultrasound and carcass traits other than CWT and FC were low to moderate and negative and ranged from −0.48 to −0.19. Overall, estimates of genetic correlations with finishing traits were similar compared to those with MWT after the effect of BCS has been removed. Adjusting MWT to a constant height generally reduced all genetic correlations but estimates were slightly higher for the carcass traits OSS and RF. Correlations among MHH and finishing traits were slightly lower but similar to those with MWT.

The trait BCS was lowly genetically correlated with all finishing traits except for a moderate correlation with PYG (0.36) and OSS (−0.30). Body condition score was positively correlated with all ultrasound traits (0.18–0.29), but mainly negative genetic correlations were estimated with carcass traits other than CWT and those ranged from −0.30 to −0.07. No genetic correlation was observed between BCS and RF.

No genetic correlation was observed between the direct genetic effect of WWT (WWT_D_) and PWG, but estimates were high with all other growth traits in the current study (0.56–0.86). Genetic correlations of WWT_D_ with ultrasound or carcass traits other than CWT (0.85) ranged from a low negative correlation with CEMA (−0.29) to a low positive correlation with IMF (0.22). The maternal component of WWT (WWT_M_) was negatively correlated with both PWG and PYG (−0.56 and −0.18) but correlations with other growth traits were high and positive (0.66–0.78). Genetic correlations among WWT_M_ and ultrasound traits were low to moderate and positive, and the greatest estimate was observed with FD_P8_ (0.33). The genetic correlation for WWT_M_ was moderate only for CWT (0.42), whereas all other traits recorded at slaughter only showed low correlations with WWT_M_ (−0.06–0.21).

Phenotypic correlations among maternal performance traits and finishing traits are presented in Table 7. Similar to correlations among finishing traits, phenotypic correlations were lower compared to genetic correlations among traits measured in herd replacements and finishing cattle. The reproductive traits RB and DtC2 were only lowly correlated with all finishing traits and ranged from −0.13 to 0.09. The traits MWT, MWT_BCS_ and MHH were moderately to highly phenotypically correlated with growth traits other than PWG but correlations with ultrasound traits were low and negative (−0.25–−0.07). Only low phenotypic correlations were observed among BCS and all finishing traits (0.08–0.18). The trait WWT was generally highly correlated with growth traits (0.72–0.88), but estimates were low for PWG (−0.27) and PYG (0.05). Almost no correlations were observed among WWT with ultrasound and carcass traits (−0.08–0.09), but a high estimate was obtained with CWT (0.51).

## 4. Discussion

### 4.1. Inheritance of Finishing Traits

Heritabilities for the growth traits YWT and W18 were high compared with published values [12,33]. The implementation of data collection as part of the BPT and the limited number of years in which data has been recorded did not allow the estimation of maternal effects on progeny performance for either YWT or W18, thus part of the variation that would be attributable to maternal genetic effects may have been partitioned into the additive-genetic component. This may explain the greater heritability for those traits compared to literature values [33,34] and this is especially relevant for YWT where the maternal impact is likely to be considerably larger compared to W18 [33,35]. Estimating genetic parameters for growth rates rather than absolute weights is a way to account for the carry over effect of the maternal contribution to the corresponding live weight. Thus, variance components were estimated for PWG and PYG in addition to the live weight traits to avoid the requirement of fitting a maternal effect in the model. Heritability estimates for both traits agree with values presented in the literature [12,36]. All variance components increased with an increase in the mean live weights as animals grow, and this has been expected [8,37]. Previous research in beef cattle has shown that height traits are generally more heritable than weight traits [12,38,39,40] and the same effect has been shown in the current study for YHH compared to either YWT or W18. Results indicate that a large proportion of the phenotype for growth traits is attributable to differences in the genotype, thus selecting animals with higher genetic merit in those traits is likely to improve growth in the next generation.

Ultrasound traits recorded on the live animal in the current study were generally heritable and variable, thus direct selection for higher genetic merit in live animal scan traits is feasible. Most previous research has evaluated live animal ultrasound scan traits primarily on an age- rather than weight-constant basis and this needs to be considered when comparing results presented in this study with literature values. Generally, heritability estimates for ultrasound traits presented here were slightly greater compared to those values presented in the literature and the adjustment factors may explain part of the discrepancy. After adjustment of the animals to a constant weight at 18 months of age, differences in EMA are likely to be primarily due to differences in muscling rather than size. Traits related to size of the animals are likely to be more heritable than those traits related to muscling ability, and this may explain the lower estimates obtained in the current study when comparing the results to an age-constant EMA without accounting for differences in weight [41]. Copping et al. [42], however, found that results were similar with and without adjustments to a constant weight. Arnold et al. [43] estimated heritabilities for weight-constant ultrasound traits in Hereford steers of 0.26 for backfat measures and 0.25 for EMA, both of which were lower compared to values obtained in the current analysis. Similarly, the pooled heritability for a weight-constant EMA obtained by Robinson et al. [3] for Australian Angus and Hereford cattle was lower (0.21) compared to the estimate in the current study (0.36). Overall, fat-related traits in the current study had greater heritabilities compared to EMA at ultrasound scanning and this agrees with the estimates presented by Kemp et al. [41] in US Angus steers.

The current study evaluated all carcass traits other than CWT on a weight-constant basis, whereas CWT was adjusted to a constant age. A similar approach has been presented by Reverter et al. [22] for genetic evaluations within Breedplan [25]. They reported heritabilities in Australian Hereford cattle for CWT (0.54), CEMA (0.38), MB (0.36) and RF (0.27) generally in the same range compared to estimates observed in the current study. Similarly, Benyshek [44] reported estimates for CWT on an age-constant basis of 0.48 for commercially farmed Hereford cattle and this is consistent with the result reported in the current study. All other carcass traits in their study have been obtained on an age- and weight-constant basis. The heritability estimate of CEMA in the current study agrees with the estimate obtained by Benyshek [44] of 0.41 but both estimates for RF (0.51) and MB (0.46) were greater compared with the results from the current analysis. Both heritability estimates for MB (0.35) and CEMA (0.46) presented by Arnold et al. [43] on a weight-constant basis are consistent with current estimates. A study conducted by Cundiff et al. [45] indicates that heritabilities of carcass traits obtained on a weight-constant basis differ only slightly from those adjusted to a constant age. Heritabilities were, however, slightly lower for the longissimus dorsi area (CEMA) following weight adjustments and this is also reflected in the phenotypic variance. A similar trend has been reported by Kemp et al. [41]. Those results support the assertion that weight adjustments remove part of the variation that is explained through size of the animal as previously highlighted for ultrasound traits, and this is reflected in the lower heritability. Börner et al. [46], however, found similar heritabilities for CEMA adjusted to a constant age. No heritability was estimable for MC in the current study and estimates were low for FC and OSS and this may be attributable to low variation of the observed carcass quality traits. Research, however, has generally shown higher values for FC of 0.33 [47].

### 4.2. Correlations among Traits Measured in Finishing Cattle

Generally, genetic correlations among all growth traits were high, indicating that animals with favourable genotypes for one trait also have favourable genotypes for the other traits and this has also been observed by previous researchers [9,34,40]. Similarly, the fat-related ultrasound scan traits IMF, FD_P8_ and FD_RIB_ were highly correlated amongst themselves. Thus, genetic progress in one trait can lead to a correlated response in other scan traits in the same direction, and this is in agreement with the literature [3,41]. However, almost no genetic relationship existed between EMA and FD_RIB_ (0.06) or between EMA and FD_P8_ (0.06) at ultrasound scanning, which was in contrast with the moderate genetic correlation between backfat and longissimus muscle area (0.39) presented by Arnold et al. [43]. Genetic correlations among weight-constant carcass traits were generally low to moderate. The trait CEMA was only lowly correlated to both fat-related carcass traits MB and RF in the current study, and this aligns with the observation in ultrasound scan traits. Thus, breeders wanting to improve muscling ability in finishing cattle can achieve this without reducing marbling of the carcass. Börner et al. [46] found equally low correlations among the age-constant carcass traits CEMA and MB (0.18) as well as CEMA and RF (−0.14). They, however, reported no correlation among MB and RF and this is in contrast to the moderate genetic correlation (0.38) found in this study.

Several researchers have examined genetic relationships between ultrasound traits and the corresponding traits recorded on the carcass at slaughter. Carcass traits measured via ultrasound scanning in live animals are used as indicator traits for carcass merit, allowing an early, more simplified prediction compared to measuring the actual economically relevant traits at slaughter [48]. Kemp et al. [41] studied the relationship at both a constant age and weight. Aligning with the results found in the current study, their results have shown that the genetic correlation among the muscling traits EMA and CEMA (0.58) was lower compared to the correlations among corresponding fat-related traits. The estimated correlation between FD_RIB_ and RF of 0.86 reported by Kemp et al. [41] was consistent with the value obtained in the current analysis (0.95). The correlation among the intramuscular fat traits IMF and MB by Kemp et al. [41] of 0.94, however, was higher compared to the value in the current analysis (0.70), whereas MacNeil and Northcutt [48] presented results for the genetic correlation among IMF and MB in US Angus cattle in the same range (0.52–0.84). Generally, correlations were high among corresponding traits measured at ultrasound scanning and at slaughter, such that particularly FD_RIB_ is likely to be an accurate predictor of RF recorded on the carcass at slaughter and can be used as a selection tool for genetic improvement [3,41]. Similarly, genetic gain in MB is likely to be achieved when improving the genotype for finishing cattle via IMF measured at ultrasound scanning. However, at a genetic correlation of 0.70, there are limits on the accuracy in identifying animals with superior genetics for MB on the carcass when using IMF as a correlated predictor and the same is true for EMA and CEMA with a genetic correlation of 0.53.

### 4.3. Associations among Maternal and Finishing Performance

Results from this study indicate that RB in female herd replacements has only limited genetic correlations with growth and weight traits in finishing cattle. The same has been observed for DtC2 which describes a similar trait to RB using a continuous as opposed to binary measure of reproductive success. Increased reproductive performance in female herd replacements in terms of first-calving heifers getting back in calf early in their second mating season had a positive genetic correlation with fat traits in finishing animals at either live animal ultrasound scanning or at slaughter. Thus, genetic progress in the fat content of finishing cattle is likely to move the average of the replacement herd towards animals with higher pregnancy outcomes. Wolcott et al. [4] examined the genetic relationship among reproductive traits in females and finishing performance in steers. This research was conducted for the Australian Brahman and Tropical Composite breeds and those are likely to differ from estimates obtained for British breeds such as Hereford and Angus particularly in terms of reproduction. Genetic correlations presented in their research, however, tend to align with estimates from the current study. Wolcott et al. [4] reported a positive genetic correlation between MB and pregnancy rate of 0.20 and between MB and days to calving from −0.13 to −0.11 and this is consistent with the correlations of 0.22 between MB and RB and −0.29 between MB and DtC2 found in the current study. The moderate negative genetic correlation among RB and CEMA indicates that a reduction in pregnancy outcomes in 2-year-old cows is likely when improving the genetic merit for muscling ability in finishing cattle. The large standard error for this correlation, however, implies that further research is required to confirm this assertion. The same applies to correlations with FC and OSS and this may be attributable to low heritabilities of both carcass quality traits.

Mature cow weight was highly genetically correlated with growth and other weight traits such as YWT, W18 and CWT and this has been previously reported in the literature [1,11]. Thus, balancing selection strategies is required to improve growth and carcass traits in finishing cattle while at the same time reducing the size of mature cows to minimise energy requirements for maintenance. High genetic correlations, however, may limit the opportunity to identify animals with high growth but moderate mature size genotypes. The negative genetic correlations of MWT with either EMA or CEMA indicates that improving the genetic merit of muscularity in finishing animals at the same slaughter endpoint is likely to move the average of the replacement herd towards cow that exhibit overall lower MWT. Similarly, moderate and negative genetic correlations were observed for MWT with fat traits measured either via ultrasound on live animals or at slaughter. Thus, selection for higher fat content in finishing animals is likely to reduce MWT. Overall, relationships were consistent to those with MWT after the phenotypic association with BCS or HH has been removed. Similarly, MHH behaved in a similar manner when comparing among genetic correlations with all finishing traits.

Body condition score was only lowly genetically correlated to all finishing traits in the current study. Contrary to common belief among breeders, the results have shown that the fat content in finishing animals at either ultrasound scanning or on the carcass is not strongly related to the energy reserves in mature cows as indicated by BCS. This indicates that if breeders want to increase the BCS of their cows by using fat traits in finishing cattle as a selection tool, a large increase in the genetic merit of ultrasound and carcass fat traits would be required for only a small change in BCS. Generally, a greater response in BCS is likely when including a direct measure of BCS in the selection program and breeders may improve BCS in their cows without greatly impacting the fattening potential of the finishing herd. Practical application may, however, be limited by the time delay in measuring mature cows and/or the number of cows available for selection.

Genetic correlations among WWT_D_ and growth traits were generally high, and this agrees with literature values [9,40]. All ultrasound and carcass traits other than CWT were only lowly genetically correlated with WWT_D_ and this indicates only a limited impact on fat content and muscularity for finishing cattle at a constant weight when aiming for genetic progress in WWT_D_. Improving maternal genetic ability in terms of calf weaning weight tends to lead to a correlated response in weight and height traits of finishing animals in the same direction but those animals are less likely to exhibit greater genetic merit for growth rates until W18. Overall, WWT_M_ was only lowly genetically correlated with carcass traits other than CWT and correlations were only slightly higher with those traits measured at ultrasound scanning. Thus, antagonistic effects on any traits related to performance in finishing cattle are unlikely when improving the genetic merit for WWT_M_. The genetic correlations among WWT_M_ and the carcass traits CWT, RF and MB agree with the estimates presented by Crews et al. [49] in Canadian Charolais cattle of 0.27, −0.02 and −0.07, respectively. Estimates presented by Splan et al. [50] were, however, larger for all carcass traits and this may be attributable to the adjustment method of carcass traits to a constant age rather than weight.

## 5. Conclusions

To optimize the efficiency of the entire beef herd, knowledge of the relationships among traits that affect the maternal and the finishing operation is required. Generally, results indicate that genetic correlations exist among key maternal performance traits and finishing traits and those need to be taken into account to avoid potential antagonisms when developing selection programs and for genetic evaluations. Balanced selection is required to enhance production in both systems without compromising performance in correlated traits. Genetic correlations indicate that there is potential to reduce the fat content in either live animals at ultrasound scanning or at slaughter with only minor changes in body energy reserves in mature cows, but this may have a detrimental effect on the genetic merit for reproductive performance in 2-year-old cows. Thus, it is important to consider traits in a multi-trait context to achieve genetic gain in the entire system. Results indicate that it is possible to identify animals with favourable genotypes for finishing and maternal performance when measures are available for both trait groups.

## Figures and Tables

**Table 1:** Contemporary group (CG) definitions for maternal, growth, ultrasound and carcass traits and number (*n*) of CGs.

Trait	CG	*n* of CGs
Maternal traits		
RB; DtC2	Herd × recording date × birth year × management group at recording	12; 13
MWT; MBCS	Herd × recording date × management group	261; 259
MHH	Herd × recording date	18
WWT	Herd × sex × recording date × birth management group × weaning management group	200
Growth traits		
PWG/YWT	Herd × sex × recording date × birth group × weaning group × yearling group	239
PYG/W18	Herd × sex × recording date × birth group × weaning group × yearling group × W18 group	224
YHH	Herd × sex × recording date	43
Ultrasound traits		
EMA/IMF/FD_P8_/FD_RIB_	Herd × sex × recording date × birth group × weaning group × yearling group × W18 group	214–220
Carcass traits		
CWT/CEMA/MB/FC/MC/OSS/RF	Herd × sex × recording date × birth group × weaning group × yearling group × W18 group × slaughter group	105–209

RB = rebreeding; DtC2 = days to conception; MWT = mature cow live weight; BCS = mature cow body condition score; MHH = mature cow hip height; WWT = weaning weight of calves; PWG = post-weaning gain; YWT = yearling weight; PYG = post-yearling gain; W18 = 18 month weight; YHH = yearling hip height; EMA = eye-muscle area (ultrasound); IMF = intramuscular fat (ultrasound); FD_P8_ = P8 fat depth (ultrasound); FD_RIB_ = rib fat depth (ultrasound); CWT = hot carcass weight; CEMA = eye-muscle area (carcass); MB = marbling (carcass); FC = fat colour (carcass); MC = meat colour (carcass); OSS = ossification (carcass); RF = rib fat depth (carcass).

**Table 2:** Trait abbreviations (Abb.), numbers (*n*) of records, individual animals, sires and dams, range of measurements, means and standard deviations (SD) after adjustments and scaling.

Abb.	Unit	*n* of Records		*n* of Individual Records	*n* of Dams	*n* of Sires	Range	Mean (SD)
		Females	Males					
Maternal traits								
RB	%	1272	-	1272	1107	227	-	91.6
DtC2	Days	1303	-	1303	1138	230	0–91	25.8 (21.3)
MWT	kg	37,110	-	4660	897	195	408–728	562.3 (47.3)
MWT_BCS_	kg	37,098	-	4660	897	195	411–668	535.1 (39.5)
MWT_HH_	kg	4953	-	3447	817	185	440–703	572.4 (40.3)
BCS	Score	37,111	-	4662	897	195	3–10	6.9 (1.0)
MHH	cm	5172	-	3552	857	186	118–143	130.4 (4.0)
WWT	kg	3740	3797	7537	4102	406	110–338	226.4 (32.1)
Growth traits								
PWG	kg	2937	3118	6055	3396	376	−68–161	47.0 (37.6)
YWT	kg	2964	3149	6113	3420	376	152–439	276.3 (38.7)
PYG	kg	2020	1879	3899	2482	296	19–279	149.8 (39.3)
W18	kg	2044	1899	3943	2501	296	295–639	431.7 (50.2)
YHH	cm	2993	2090	5083	3053	373	94–133	115.8 (4.9)
Ultrasound traits								
EMA	cm^2^	2049	1893	3942	2498	296	45–82	63.2 (5.0)
IMF	%	2040	1904	3944	2501	296	0–9	3.2 (1.7)
FD_P8_	mm	2025	1902	3927	2489	296	1–11	5.1 (1.5)
FD_RIB_	mm	2023	1900	3923	2496	296	1–7	3.5 (1.0)
Carcass traits								
CWT	kg	351	1636	1987	1548	248	231–416	315.9 (26.4)
CEMA	cm^2^	228	960	1188	1025	170	52–98	74.6 (6.7)
MB	Score	352	1626	1978	1539	248	100–741	350.5 (88.5)
FC	Score	345	1539	1884	1483	248	0–5	2.5 (0.8)
MC	Score	350	1505	1855	1459	248	1–7	3.1 (0.7)
OSS	Score	354	1601	1955	1518	246	104–201	150.3 (13.6)
RF	mm	354	1615	1969	1532	247	0–17	5.4 (2.6)

RB = rebreeding; DtC2 = days to conception; MWT = mature cow live weight; MWT_BCS_ = MWT adjusted for body condition score; MWT_HH_ = MWT adjusted for hip height; BCS = mature cow body condition score; MHH = mature cow hip height; WWT = weaning weight of calves; PWG = post-weaning gain; YWT = yearling weight; PYG = post-yearling gain; W18 = 18 month weight; YHH = yearling hip height; EMA = eye-muscle area (ultrasound); IMF = intramuscular fat (ultrasound); FD_P8_ = P8 fat depth (ultrasound); FD_RIB_ = rib fat depth (ultrasound); CWT = hot carcass weight; CEMA = eye-muscle area (carcass); MB = marbling (carcass); FC = fat colour (carcass); MC = meat colour (carcass); OSS = ossification (carcass); RF = rib fat depth (carcass).

**Table 3:** Fixed effects and random effects for each trait included in the variance component analysis.

	Fixed Effects					Random Effects ^3^			
	Age ^1^	Age of Dam ^2^	Breed of Animal ^1^	Heterosis ^1^	CG	a	m	me	pe
Maternal traits									
RB	√	√	√	√	√	√			
DtC2	√	√	√	√	√	√			
MWT/MWT_BCS_/MWT_HH_/BCS/MHH			√	√	√	√			√
WWT		√	√	√	√	√	√	√	
Growth traits									
PWG/YWT/PYG/W18/YHH		√	√	√	√	√			
Ultrasound traits									
EMA/IMF/FD_P8_/FD_RIB_		√	√	√	√	√			
Carcass traits									
CWT/CEMA/MB/FC/MC/OSS/RF			√	√	√	√			

RB = rebreeding; DtC2 = days to conception; MWT = mature cow live weight; MWT_BCS_ = MWT adjusted for body condition score; MWT_HH_ = MWT adjusted for hip height; BCS = mature cow body condition score; MHH = mature cow hip height; WWT = weaning weight of calves; PWG = post-weaning gain; YWT = yearling weight; PYG = post-yearling gain; W18 = 18 month weight; YHH = yearling hip height; EMA = eye-muscle area (ultrasound); IMF = intramuscular fat (ultrasound); FD_P8_ = P8 fat depth (ultrasound); FD_RIB_ = rib fat depth (ultrasound); CWT = hot carcass weight; CEMA = eye-muscle area (carcass); MB = marbling (carcass); FC = fat colour (carcass); MC = meat colour (carcass); OSS = ossification (carcass); RF = rib fat depth (carcass).^1^ Covariate; ^2^ Class effect; ^3^ a = additive-genetic effect; m = maternal genetic effect; me = maternal permanent environmental effect; pe = permanent environmental effect.

**Table 4:** Variance components ($\sigma_{A}^{2}$ = additive genetic; $\sigma_{R}^{2}$ = residual), heritability estimates (h^2^) and their standard errors (±SE) from uni- and bivariate analysis for growth, ultrasound and carcass traits in New Zealand beef cattle.

Trait	$\sigma_{A}^{2}$	$\sigma_{R}^{2}$	h^2^
Growth traits			
PWG	95.50	180.69	0.35 ± 0.04
YWT	339.81	301.71	0.53 ± 0.04
PYG	119.16	242.58	0.33 ± 0.05
W18	468.59	459.58	0.50 ± 0.06
YHH	6.97	5.75	0.55 ± 0.04
Ultrasound traits			
EMA	5.84	10.26	0.36 ± 0.05
IMF	0.91	0.84	0.52 ± 0.05
FD_P8_	1.26	0.92	0.58 ± 0.05
FD_RIB_	0.53	0.45	0.54 ± 0.05
Carcass traits			
CWT	134.66	151.36	0.47 ± 0.07
CEMA	13.91	18.79	0.43 ± 0.11
MB	2122.33	3287.59	0.39 ± 0.08
FC	0.03	0.35	0.07 ± 0.06
MC ^1^	0.00	0.51	0.00
OSS	24.21	178.87	0.12 ± 0.06
RF	2.01	5.18	0.28 ± 0.07

PWG = post-weaning gain; YWT = yearling weight; PYG = post-yearling gain; W18 = 18 month weight; YHH = yearling hip height; EMA = eye-muscle area (ultrasound); IMF = intramuscular fat (ultrasound); FD_P8_ = P8 fat depth (ultrasound); FD_RIB_ = rib fat depth (ultrasound); CWT = hot carcass weight; CEMA = eye-muscle area (carcass); MB = marbling (carcass); FC = fat colour (carcass); MC = meat colour (carcass); OSS = ossification (carcass); RF = rib fat depth (carcass). ^1^ Convergence was not achieved from bivariate model; results were obtained from univariate model.

**Table 5:** Genetic (SE; below diagonal) and phenotypic correlations (SE; above diagonal) among finishing traits in New Zealand beef cattle.

	PWG	YWT	PYG	W18	YHH	EMA	IMF	FD_P8_	FD_RIB_	CWT	CEMA	MB	FC	OSS	RF
PWG		0.46 (0.01)	−0.13 (0.02)	0.29 (0.02)	0.18 (0.02)	−0.03 (0.02)	0.04 (0.02)	0.07 (0.02)	0.03 (0.02)	0.18 (0.02)	−0.04 (0.03)	0.04 (0.03)	−0.01 (0.03)	0.02 (0.02)	0.00 (0.03)
YWT	0.16 (0.06)		−0.05 (0.02)	0.77 (0.01)	0.67 (0.01)	0.01 (0.02)	0.08 (0.02)	0.08 (0.02)	0.07 (0.02)	0.56 (0.02)	−0.09 (0.04)	−0.04 (0.03)	−0.01 (0.03)	0.01 (0.03)	0.01 (0.03)
PYG	0.10 (0.10)	0.21 (0.09)		0.60 (0.01)	0.15 (0.02)	−0.05 (0.02)	0.00 (0.02)	−0.06 (0.02)	−0.07 (0.02)	0.44 (0.02)	0.02 (0.03)	−0.07 (0.03)	−0.02 (0.03)	−0.03 (0.03)	−0.10 (0.03)
W18	0.18 (0.08)	0.88 (0.02)	0.68 (0.06)		0.60 (0.01)	−0.04 (0.02)	0.06 (0.02)	0.02 (0.02)	0.01 (0.02)	0.71 (0.01)	−0.03 (0.04)	−0.07 (0.03)	−0.02 (0.03)	−0.01 (0.03)	−0.06 (0.03)
YHH	0.08 (0.07)	0.78 (0.03)	0.37 (0.09)	0.70 (0.04)		−0.09 (0.02)	−0.03 (0.02)	−0.05 (0.02)	−0.04 (0.02)	0.50 (0.02)	−0.09 (0.04)	−0.06 (0.03)	0.02 (0.03)	−0.02 (0.03)	−0.04 (0.03)
EMA	−0.28 (0.09)	−0.08 (0.09)	0.00 (0.11)	−0.12 (0.09)	−0.26 (0.08)		0.22 (0.02)	0.15 (0.02)	0.16 (0.02)	0.04 (0.02)	0.18 (0.03)	0.05 (0.02)	−0.01 (0.02)	0.04 (0.02)	0.07 (0.02)
IMF	0.00 (0.08)	0.14 (0.07)	−0.16 (0.09)	−0.03 (0.08)	−0.25 (0.07)	0.36 (0.08)		0.46 (0.01)	0.55 (0.01)	0.06 (0.02)	0.07 (0.03)	0.28 (0.02)	0.03 (0.03)	0.04 (0.03)	0.25 (0.02)
FD_P8_	0.06 (0.08)	0.09 (0.07)	−0.22 (0.09)	−0.11 (0.08)	−0.14 (0.07)	0.06 (0.09)	0.68 (0.05)		0.72 (0.01)	−0.07 (0.02)	−0.02 (0.03)	0.22 (0.02)	0.03 (0.02)	0.02 (0.02)	0.32 (0.02)
FD_RIB_	−0.01 (0.08)	0.06 (0.07)	−0.25 (0.09)	−0.07 (0.08)	−0.20 (0.07)	0.06 (0.09)	0.76 (0.04)	0.85 (0.03)		−0.05 (0.02)	−0.01 (0.03)	0.22 (0.02)	0.03 (0.02)	0.06 (0.02)	0.35 (0.02)
CWT	0.09 (0.10)	0.74 (0.05)	0.66 (0.08)	0.84 (0.04)	0.65 (0.06)	0.14 (0.11)	0.07 (0.09)	−0.06 (0.09)	−0.05 (0.09)		0.03 (0.03)	−0.04 (0.03)	−0.02 (0.03)	−0.02 (0.03)	−0.03 (0.03)
CEMA	−0.01 (0.15)	−0.32 (0.14)	0.06 (0.16)	−0.16 (0.15)	−0.39 (0.14)	0.53 (0.13)	0.17 (0.13)	−0.07 (0.13)	−0.07 (0.13)	−0.12 (0.16)		0.08 (0.03)	0.05 (0.03)	0.08 (0.03)	−0.06 (0.03)
MB	0.17 (0.12)	0.06 (0.11)	−0.21 (0.13)	−0.06 (0.12)	−0.08 (0.11)	−0.11 (0.12)	0.70 (0.08)	0.39 (0.10)	0.38 (0.10)	0.09 (0.13)	0.09 (0.17)		0.07 (0.02)	0.09 (0.02)	0.24 (0.02)
FC	0.11 (0.25)	−0.32 (0.27)	−0.04 (0.27)	−0.37 (0.27)	−0.35 (0.27)	−0.34 (0.26)	−0.07 (0.24)	−0.09 (0.24)	−0.19 (0.24)	−0.63 (0.34)	0.73 (0.49)	−0.01 (0.29)		0.05 (0.02)	0.09 (0.02)
OSS	−0.06 (0.20)	0.32 (0.17)	−0.20 (0.20)	−0.03 (0.19)	−0.08 (0.18)	0.23 (0.19)	0.10 (0.18)	−0.06 (0.18)	0.14 (0.17)	−0.06 (0.21)	−0.30 (0.26)	0.31 (0.22)	0.07 (0.45)		0.04 (0.02)
RF	0.10 (0.14)	0.18 (0.13)	−0.25 (0.15)	−0.07 (0.14)	0.02 (0.13)	0.12 (0.14)	0.72 (0.11)	0.66 (0.11)	0.95 (0.12)	0.03 (0.15)	−0.18 (0.19)	0.38 (0.15)	0.08 (0.33)	0.27 (0.26)	

PWG = post-weaning gain; YWT = yearling weight; PYG = post-yearling gain; W18 = 18 month weight; YHH = yearling hip height; EMA = eye-muscle area (ultrasound); IMF = intramuscular fat (ultrasound); FD_P8_ = P8 fat depth (ultrasound); FD_RIB_ = rib fat depth (ultrasound); CWT = hot carcass weight; CEMA = eye-muscle area (carcass); MB = marbling (carcass); FC = fat colour (carcass); MC = meat colour (carcass); OSS = ossification (carcass); RF = rib fat depth (carcass).

**Table 6:** Genetic correlations (SE) among maternal and finishing traits in New Zealand beef cattle.

	RB	DtC2	MWT	MWT_BCS_	MWT_HH_	BCS	MHH	WWT_D_	WWT_M_
PWG	0.11 (0.22)	−0.31 (0.19)	0.45 (0.05)	0.39 (0.05)	0.43 (0.08)	0.25 (0.06)	0.13 (0.06)	0.04 (0.12)	−0.56 (0.11)
YWT	0.14 (0.20)	−0.26 (0.17)	0.64 (0.04)	0.66 (0.04)	0.26 (0.07)	0.12 (0.05)	0.43 (0.05)	0.86 (0.03)	0.78 (0.10)
PYG	0.22 (0.25)	0.12 (0.22)	0.86 (0.05)	0.84 (0.05)	0.64 (0.10)	0.36 (0.08)	0.57 (0.07)	0.56 (0.12)	−0.18 (0.12)
W18	0.32 (0.24)	−0.19 (0.19)	0.92 (0.04)	0.93 (0.03)	0.53 (0.09)	0.27 (0.06)	0.64 (0.05)	0.80 (0.05)	0.66 (0.15)
YHH	0.18 (0.20)	−0.33 (0.17)	0.62 (0.04)	0.67 (0.04)	−0.04 (0.07)	0.02 (0.05)	0.80 (0.04)	0.67 (0.06)	0.77 (0.12)
EMA	0.11 (0.25)	−0.13 (0.20)	−0.29 (0.06)	−0.36 (0.05)	−0.22 (0.09)	0.20 (0.07)	−0.22 (0.07)	0.08 (0.13)	0.19 (0.11)
IMF	0.35 (0.25)	−0.42 (0.19)	−0.19 (0.05)	−0.24 (0.05)	−0.06 (0.08)	0.18 (0.06)	−0.25 (0.06)	0.22 (0.11)	0.14 (0.10)
FD_P8_	0.31 (0.25)	−0.35 (0.19)	−0.28 (0.05)	−0.39 (0.04)	−0.13 (0.08)	0.29 (0.06)	−0.31 (0.05)	−0.18 (0.12)	0.33 (0.10)
FD_RIB_	0.39 (0.25)	−0.37 (0.19)	−0.32 (0.05)	−0.41 (0.04)	−0.19 (0.08)	0.25 (0.06)	−0.32 (0.05)	−0.10 (0.12)	0.26 (0.10)
CWT	0.13 (0.26)	−0.08 (0.22)	0.82 (0.06)	0.82 (0.05)	0.40 (0.11)	0.24 (0.08)	0.65 (0.07)	0.85 (0.06)	0.42 (0.14)
CEMA	−0.46 (0.37)	0.21 (0.31)	−0.29 (0.10)	−0.23 (0.10)	−0.06 (0.16)	−0.07 (0.12)	−0.21 (0.12)	−0.29 (0.18)	−0.04 (0.20)
MB	0.22 (0.30)	−0.29 (0.26)	−0.35 (0.08)	−0.35 (0.08)	−0.34 (0.13)	−0.10 (0.10)	−0.17 (0.09)	0.01 (0.15)	−0.06 (0.16)
FC	−0.32 (0.59)	0.01 (0.50)	−0.52 (0.29)	−0.55 (0.28)	−0.48 (0.33)	0.13 (0.24)	−0.39 (0.27)	n.e.	n.e.
OSS	−0.50 (0.44)	0.19 (0.38)	−0.48 (0.16)	−0.45 (0.15)	−0.59 (0.23)	−0.30 (0.20)	−0.28 (0.16)	0.12 (0.22)	0.21 (0.27)
RF	0.36 (0.35)	−0.59 (0.29)	−0.40 (0.10)	−0.43 (0.09)	−0.49 (0.15)	0.02 (0.12)	−0.28 (0.10)	0.03 (0.16)	0.00 (0.19)

RB = rebreeding; DtC2 = days to conception; MWT = mature cow live weight; MWT_BCS_ = MWT adjusted for body condition score; MWT_HH_ = MWT adjusted for hip height; BCS = mature cow body condition score; MHH = mature cow hip height; WWT = weaning weight of calves; PWG = post-weaning gain; YWT = yearling weight; PYG = post-yearling gain; W18 = 18 month weight; YHH = yearling hip height; EMA = eye-muscle area (ultrasound); IMF = intramuscular fat (ultrasound); FD_P8_ = P8 fat depth (ultrasound); FD_RIB_ = rib fat depth (ultrasound); CWT = hot carcass weight; CEMA = eye-muscle area (carcass); MB = marbling (carcass); FC = fat colour (carcass); MC = meat colour (carcass); OSS = ossification (carcass); RF = rib fat depth (carcass). n.e. = non-estimable.

**Table 7:** Phenotypic correlations (SE) among maternal and finishing traits in New Zealand beef cattle.

	RB	DtC2	MWT	MWT_BCS_	MWT_HH_	BCS	MHH	WWT
PWG	0.08 (0.03)	−0.11 (0.03)	0.19 (0.03)	0.18 (0.03)	0.11 (0.03)	0.08 (0.03)	0.20 (0.03)	−0.27 (0.02)
YWT	0.04 (0.03)	−0.07 (0.03)	0.35 (0.02)	0.41 (0.02)	0.28 (0.03)	0.09 (0.03)	0.42 (0.03)	0.88 (0.01)
PYG	0.06 (0.03)	−0.04 (0.03)	0.39 (0.03)	0.43 (0.03)	0.31 (0.03)	0.14 (0.04)	0.35 (0.03)	0.05 (0.02)
W18	0.05 (0.03)	−0.05 (0.03)	0.57 (0.02)	0.64 (0.02)	0.40 (0.03)	0.18 (0.03)	0.53 (0.02)	0.72 (0.02)
YHH	0.01 (0.03)	−0.03 (0.03)	0.37 (0.02)	0.43 (0.02)	0.07 (0.03)	0.09 (0.03)	0.61 (0.02)	0.72 (0.02)
EMA	0.03 (0.03)	−0.05 (0.03)	−0.11 (0.03)	−0.19 (0.03)	−0.09 (0.04)	0.08 (0.04)	−0.11 (0.04)	0.06 (0.02)
IMF	0.08 (0.03)	−0.08 (0.03)	−0.07 (0.03)	−0.13 (0.03)	0.04 (0.04)	0.08 (0.03)	−0.08 (0.03)	0.08 (0.02)
FD_P8_	0.09 (0.03)	−0.11 (0.03)	−0.14 (0.03)	−0.25 (0.03)	0.03 (0.04)	0.16 (0.03)	−0.18 (0.03)	0.09 (0.02)
FD_RIB_	0.08 (0.03)	−0.13 (0.03)	−0.16 (0.03)	−0.25 (0.03)	−0.02 (0.04)	0.09 (0.04)	−0.18 (0.03)	0.08 (0.02)
CWT	-	-	-	-	-	-	-	0.51 (0.03)
CEMA	-	-	-	-	-	-	-	−0.06 (0.04)
MB	-	-	-	-	-	-	-	−0.08 (0.03)
FC	-	-	-	-	-	-	-	n.e.
OSS	-	-	-	-	-	-	-	−0.01 (0.03)
RF	-	-	-	-	-	-	-	−0.01 (0.03)

RB = rebreeding; DtC2 = days to conception; MWT = mature cow live weight; MWT_BCS_ = MWT adjusted for body condition score; MWT_HH_ = MWT adjusted for hip height; BCS = mature cow body condition score; MHH = mature cow hip height; WWT = weaning weight of calves; PWG = post-weaning gain; YWT = yearling weight; PYG = post-yearling gain; W18 = 18 month weight; YHH = yearling hip height; EMA = eye-muscle area (ultrasound); IMF = intramuscular fat (ultrasound); FD_P8_ = P8 fat depth (ultrasound); FD_RIB_ = rib fat depth (ultrasound); CWT = hot carcass weight; CEMA = eye-muscle area (carcass); MB = marbling (carcass); FC = fat colour (carcass); MC = meat colour (carcass); OSS = ossification (carcass); RF = rib fat depth (carcass). No phenotypic covariance exists for those correlations indicated by “-” as traits were not measured within the same animal. n.e. = non-estimable.

## Data Availability

Third-Party Data. Restrictions apply to the availability of these data. Data were obtained from Beef + Lamb New Zealand Genetics.
